# Pneumatosis cystoides intestinalis of the ascending colon related to acarbose treatment: a case report

**DOI:** 10.4076/1752-1947-3-9216

**Published:** 2009-09-08

**Authors:** Yilin Vogel, Nikolaus J Buchner, Michael Szpakowski, Andrea Tannapfel, Bernhard F Henning

**Affiliations:** 1 Department of Internal Medicine I, Marienhospital Herne, Ruhr-University Bochum, Hölkeskampring 40, 44625 Herne, Germany; 2 Department of Radiology, Marienhospital Herne, Ruhr-University Bochum, Bochum, Hölkeskampring 40, 44625 Herne, Germany; 3 Department of Pathology, Berufsgenossenschaftliches Universitätsklinikum Bergmannsheil, Ruhr-University Bochum, Bürkle de la Camp-Platz 1, 44789 Bochum, Germany

## Abstract

**Introduction:**

Pneumatosis cystoides intestinalis is characterized by the presence of multiple gas-filled cysts in the intestinal wall, the submucosa and/or subserosa of the intestine. The term pneumatosis cystoides coli is synonymous with pneumatosis cystoides intestinalis when the disorder is limited to the colon. It is a secondary finding caused by a wide variety of underlying gastrointestinal or extragastrointestinal diseases but rarely occurs in the course of treatment with an α-glucosidase inhibitor. This is the first report of pneumatosis cystoides intestinalis after 12 years of treatment with the α-glucosidase inhibitor acarbose.

**Case presentation:**

A 65-year-old Caucasian German woman was referred to our hospital for hemicolectomy. She had been treated for type 2 diabetes mellitus with an α-glucosidase inhibitor (acarbose, 150 mg daily) for 12 years. Three months before referral, she had complained of left abdominal pain. 'Polyposis coli' in the ascending colon and diverticulosis were diagnosed. Colonoscopy and computed tomography scans of the abdomen were repeated and revealed pneumatosis cystoides coli located in the ascending colon, whereas diverticulosis of the sigmoid colon was confirmed. Histological examination of a biopsy specimen only showed colon mucosa. After discontinuing administration of the α-glucosidase inhibitor for 3 months and on repeated colonoscopy, the polypoid lesions had completely disappeared.

**Conclusion:**

This case illustrates that pneumatosis cystoides coli can be a source of diagnostic confusion. Pneumatosis cystoides coli must be considered in the initial differential diagnosis of patients especially in the presence of multiple colonic polypoid lesions. It is important to take pneumatosis cystoides intestinalis into consideration when prescribing α-glucosidase inhibitors to patients with diabetes who have diabetic autonomic neuropathy with decreased intestinal motility, or to patients taking steroids.

## Introduction

Pneumatosis cystoides intestinalis (PCI), defined as the presence of gas inside the intestinal wall, may be located in any part of the gastrointestinal tract. In PCI, gas is found in a linear or cystic form in the subserosa or submucosa [1]. The subserous cysts are most frequently found in the small bowel while the submucous localizations are predominantly seen in the colonic wall [2]. PCI is a secondary finding caused by a wide variety of underlying gastrointestinal or extragastrointestinal diseases such as autoimmune (scleroderma, dermatomyositis), inflammatory (inflammatory bowel disease), or infectious diseases (*Clostridium difficile*, HIV), pulmonary disease (chronic obstructive pulmonary disease), drugs (corticosteroids, immunosuppressive therapy), and trauma (blunt abdominal trauma, endoscopy).

In most cases, PCI presents with mild gastrointestinal symptoms. Symptoms include diarrhea, mucus discharge, rectal bleeding and constipation [3]. The diagnosis is suspected by endoscopy and confirmed by computed tomography (CT) and histological examination of biopsy specimens. The endoscopic differential diagnosis of more common diseases can be difficult. In the colon, gas-filled cysts are often misdiagnosed as polyps, carcinoma, lymphoma, and colitis cystica profunda.

Patients may be treated with oxygen and/or antibiotics. Urgent surgical intervention is only required in rare cases of PCI with perforation and necrotic bowel.

## Case presentation

A 65-year-old Caucasian German woman complained of left abdominal pain 3 months before referral. Under the suspected diagnosis of acute sigmoid diverticulitis, she had received nonspecific antibiotic therapy with ciprofloxacin for 5 days. Two weeks later, colonoscopy revealed numerous polypoid lesions located in the ascending colon. The histology of a biopsy specimen revealed normal colon mucosa. Nevertheless, she was referred to our hospital for hemicolectomy with the diagnosis still suspected to be polyposis coli.

Non-insulin-dependent diabetes mellitus had been diagnosed 12 years earlier. Since then, she had taken 150 mg acarbose every day. There were no other episodes of abdominal problems during that 12-year period.

Her medical history included hypertension, typhus abdominalis with ulcer 57 years previously, and hysterectomy and ovariectomy one year previously.

Physical examination showed normal blood pressure (120/60 mmHg), regular heart rate (76 beats/minute), and a body temperature of 36.6°C. Heart sounds were clear and the rhythm was regular; breath sounds were normal without rales or bronchial obstruction. The abdomen was not distended and regular peristaltic sounds were audible. Neurological examination revealed no pathological findings, in particular, no signs of diabetic polyneuropathy. Laboratory tests revealed the white blood cell count and C-reactive protein levels to be normal, but blood sugar (120 mg/dl) and HbA1c levels (6.3%) were raised.

**Figure 1:**
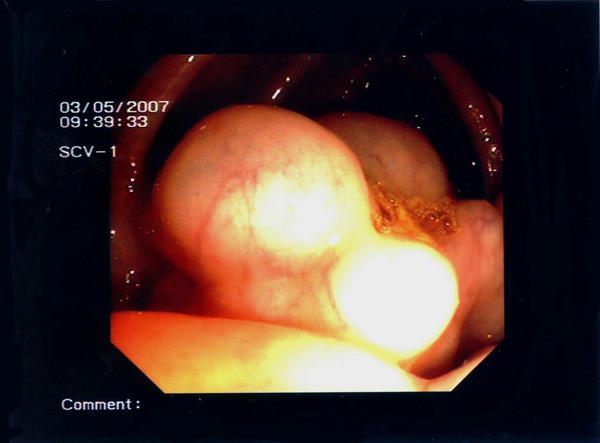
**Colonoscopy examination disclosed multiple polypoid lesions that were covered with inconspicuous mucosa with superficial vessels in the area of the ascending colon**.

**Figure 2:**
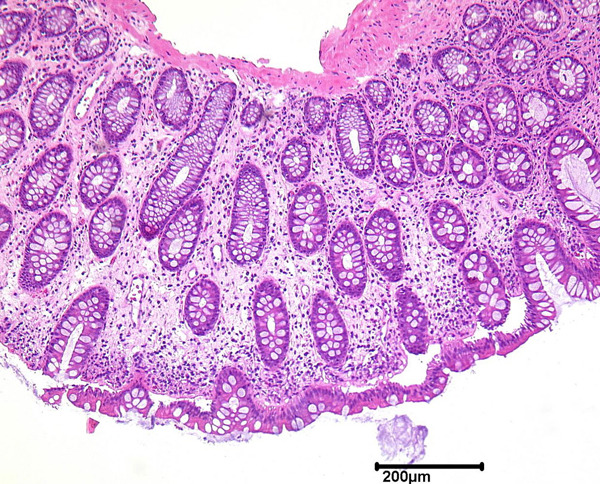
**Muscularis mucosae in pneumatosis cystoides coli from the ascending colon**.

**Figure 3:**
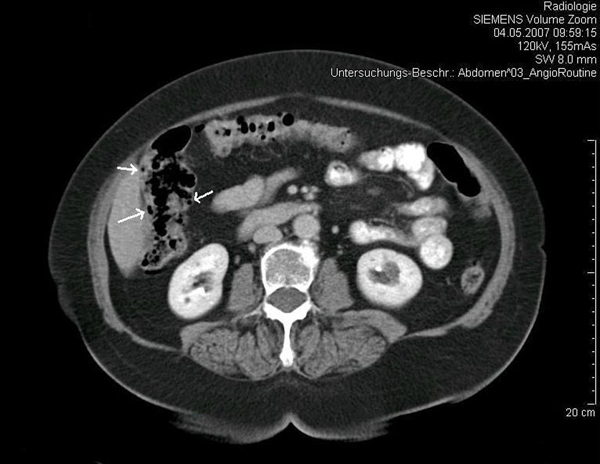
**Abdominal computed tomography scan**. Arrows show intramural air in the ascending colon.

We repeated a colonoscopy and it revealed multiple polypoid formations of varying sizes (1-3 cm) in the ascending colon, covered by normal mucosa with superficial vessels (Figure 1), and diverticulosis of the sigmoid colon. After biopsy, the cysts collapsed and disappeared. Furthermore, only colon mucosa was found in the biopsy specimens (Figure 2). While X-ray film of the abdomen did not reveal any pathological findings, CT confirmed conspicuous gas bubbles in the ascending colon (Figure 3).

At that time, we stopped the acarbose treatment. Whilst a diabetes diet was continued, neither further oral antidiabetics nor insulin were required to control diabetes mellitus. Additionally, the patient was treated with oxygen for 7 days (3 L/minute intranasally). After having discontinued the acarbose treatment for 3 months, the gas-filled cysts disappeared completely, as demonstrated by colonoscopy. Fortunately, the patient had not undergone hemicolectomy for initially suspected 'polyposis coli'. The patient has remained free of abdominal symptoms for a further 19 months.

## Discussion

The pathogenesis of PCI is still unclear and several mechanisms have been postulated for its development. The mechanical theory proposes that gas diffuses into the intestinal wall from either the intestinal lumen or the pulmonary airway. The diffusion of intraluminal gas into the intestinal wall is due to increased intraluminal pressure and the presence of mucosal injury [4,5]. Additionally, gas might travel from ruptured alveoli through the mediastinum into the retroperitoneal space and find its way into the intestinal wall along perivascular spaces through the mesentery [6]. Alternatively, the bacterial theory suggests that gas-producing bacteria entering the intestinal wall through a mucosal lesion form intramural gas [7], thus forming cysts.

Acarbose, an α-glucosidase inhibitor, is a hypoglycemic agent that can suppress postprandial hyperglycemia by delaying absorption of carbohydrates in the small intestine through antagonistic as well as dose-dependent suppression of α-glucosidase (α-GI). Well-known side effects of α-GIs include flatulence and abdominal distension resulting from fermentation by intestinal bacteria that produce carbon dioxide, methane and hydrogen from unabsorbed carbohydrates [8].

Our patient did not experience abdominal distension or flatulence. Nevertheless, she probably had elevated intraluminal pressure because she had diverticulosis of the sigmoid colon, possibly as a result of elevated intraluminal pressure and reduced power of resistance of the intestinal wall. We found one report regarding pneumatosis cystoides coli (PCC) located in the sigmoid colon due to a solitary sigmoid diverticulum- the patient was treated with corticosteroids for periarteritis nodosa [9]. In our patient, diverticulosis of the sigmoid colon was most likely not responsible for PCC in the ascending colon.

The term pneumatosis cystoides coli (PCC) is synonymous with pneumatosis cystoides intestinalis (PCI) when the disorder is limited to the colon. In our patient, we suppose that PCC was basically caused by acarbose. Two months after her initial presentation, no regression of PCC was found via colonoscopy during continuation of her treatment with acarbose. However, after discontinuing treatment with acarbose for 3 months, the PCC located in the ascending colon had completely disappeared. It is assumed that, in our patient, PCC developed from a combination of the α-GI leading to elevated intraluminal pressure from increased gas volume due to bacterial overgrowth as well as mucosal damage in the ascending colon due to elevated intraluminal pressure. We can only speculate that the susceptibility to the formation of PCC was due to colon mucosal vulnerability after the patient's typhus abdominalis with ulcer about 57 years previously. There were no symptoms indicating previous episodes of PCI in this patient. We believe that ageing and its related changes to the colonic wall reached a critical point after 12 years, allowing gas invasion by intraluminal pressure. Patients with PCI after acarbose treatment are generally older, the youngest one being 53 years of age (Table 1).

**Table 1:** Eight reported cases with pneumatosis cystoides intestinalis induced by α-glucosidase inhibitor administration for diabetes mellitus (modified from [10])

Author	Age, sex	Diabetes mellitus +	Concomitant drug	α-GI, daily dose, duration	Main symptoms	Localization	Prescription of α-GI+ after onset of PCI	Outcome	Treatment
Tsujimoto *et al.*[10]	69, M	Myasthenia gravis	Steroid SU	Voglibose 0.6 mg 20 months	Increased flatus, constipation, hematochezia	Sigmoid colon	Discontinuation	Healing after 14 days	Conservative
Hisamoto *et al.*[11]	56, F	Intestinal pneumonitis	Steroid	Voglibose 0.6 mg 7 days	Dyspnea, no abdominal symptoms	Ascending colon	Discontinuation	Healing after 7 days	Conservative
Maeda *et al.*[12]	72, F	Nephrotic syndrome	Insulin Steroid Immunosuppressant	Voglibose 0.9 mg 3 years	Abdominal pain, SIRS, DIC		Discontinuation	Healing after 7 days	Conservative
Saito *et al*. [13]	53, F	Dermatomyositis	Steroid Immunosuppressant	Voglibose 0.6 mg 1 month	Nausea, flatulence	Ascending colon + descending colon	Discontinuation	Healing after 21 days	Conservative
Yanaru *et al.*[14]	61, M	Unknown	SU	Voglibose 0.6 mg 5 years	Constipation, hematochezia	Sigmoid colon	Discontinuation	Healing after 28 days	Conservative
Azami [15]	87, F	Hypothyroidism	SU	Acarbose 150 mg 1 year	Paralytic ileus, abdominal distension	Small intestine	Discontinuation	Healing after 5 days	Conservative
Hayakawa *et al.*[16]	64, F	Unknown	Insulin	Voglibose 0.6 mg 1 month	Abdominal distention, flatulence	Ascending colon + transverse colon	Discontinuation	Healing after 4 days	Conservative
Furio *et al.*[17]	64, F	Unknown	Insulin	Acarbose unknown 3 years	Diarrhea, abdominal pain, weight loss	Sigmoid colon ascending colon + descending colon caecum	Discontinuation	Healing after 15 days	Conservative
Our patient	65, F	Hypertension		Acarbose 150 mg 12 years	Left abdominal pain	Ascending colon	Discontinuation	Healing after 7 days	Conservative

Six additional cases published in the Japanese language were recently reviewed by Tsujimoto *et al*. [10]. α-GI, α-glucosidase inhibitor; M, male; F, female; SIRS, systemic inflammatory response syndrome; DIC, disseminated intravascular coagulation; SU, sulfanylurea.

In our patient, we used oxygen treatment applied by a mask for 8 hours daily for 1 week. We did not use any antibiotics. However, we do not definitely believe that oxygen therapy basically caused regression of PCC, because the patient was discharged home without oxygen therapy.

Chronic obstructive pulmonary disease is often related to the development of pneumatosis intestinalis but was excluded in this patient. Our patient did not show signs of diabetic polyneuropathy. Even though, from her medical history, she did not have constipation, we cannot exclude a diabetic autonomic neuropathy with decreased colonic motility.

A review of the international literature revealed eight cases of PCI (Table 1) associated with treatment with α-GI; seven out of the eight patients were Japanese and one was Italian. In addition, Tsujimoto *et al.* reviewed another six cases reported in the Japanese language [10]. We postulate that α-GI may be prescribed more often in Japan and/or diagnosis (via CT and colonoscopy) is reached more often in Japan than in other countries, and/or Japanese patients with PCI have more clinical symptoms (Table 1: six out of the seven Japanese cases reported). In contrast with a statistical study of 919 cases by Jamart [2] (male to female ratio: 1.9:1), only two of the eight cases reported in Table 1 were male. Tsujimoto *et al.* found a peak incidence between 52 and 87 years [10], which is higher than the 41 and 50 year range reported by Jamart [2]. The difference in peak incidence may be due to the differing ethnicity of the two groups; or it may be related to the fact that the patients all had type 2 diabetes mellitus, suggesting that PCI may take more time to develop in some people with diabetes. PCI usually affects the left side of the colon and 70% of cases involve the sigmoid colon [2,3].

The duration of α-GI treatment before presentation of PCI has been reported elsewhere as 7 days to 5 years, but it was 12 years in our patient.

## Conclusion

This case report illustrates that PCI can be a source of diagnostic confusion. PCI should be considered in the initial differential diagnosis of patients, especially in the presence of colonic multiple polypoid lesions. It is also important to take PCI into consideration when prescribing α-GI to patients who have diabetes and diabetic autonomic neuropathy or to patients taking steroids.

## Abbreviations

α-GI: α-glucosidase inhibitor; CT: computed tomography; PCC: pneumatosis cystoides coli; PCI: pneumatosis cystoides intestinalis.

## Consent

Written informed consent was obtained from the patient for publication of this case report and any accompanying images. A copy of the written consent is available for review by the Editor-in-Chief of this journal.

## Competing interests

The authors declare that they have no competing interests.

## Authors' contributions

YV conceived the case report, drafted and revised the manuscript, and reviewed the relevant literature. NJB made a substantial contribution to drafting and revision of the manuscript. MS and AT helped to interpret the radiological and histological findings and reviewed the manuscript. BFH made a substantial contribution to conception and design, interpreted the case and helped in drafting the manuscript. All authors read and approved the final manuscript.
